# Cluster analysis of longitudinal treatment patterns in patients newly diagnosed with systemic lupus erythematosus in the United States

**DOI:** 10.1186/ar4668

**Published:** 2014-09-18

**Authors:** Hong Kan, Saurabh Nagar, Jeetvan Patel, Daniel J Wallace, Charles Molta

**Affiliations:** 1 GlaxoSmithKline, Research Triangle Park, NC, USA; 2 Cedars-Sinai Medical Center, Los Angeles, CA, USA; 3 GlaxoSmithKline, King of Prussia, Philadelphia, PA, USA

## Background

Treatments for systemic lupus erythematosus (SLE) include corticosteroids (CS), antimalarials, nonsteroidal anti-inflammatory drugs, cytotoxic agents, and immunosuppressive/immunomodulatory agents. We examined treatment patterns in newly diagnosed SLE patients from a multipayer US claims database.

## Methods

This study (GSK HO-13-13054) retrospectively followed incident SLE patients' treatment for 4 years in the MarketScan commercial claims database. The earliest medical claim date with SLE diagnosis (ICD-9 code 710.0x; 1 January 2002 to 31 March 2008) was the index date. Patients were ≥18 years at index, had continuous medical and pharmacy benefits for 12 months pre index without SLE diagnosis and 48 months post index, with ≥1 SLE-related inpatient claim or ≥2 office or emergency room visits with SLE diagnosis ≥30 days apart within 12 months post index. A specialist must have made ≥1 SLE diagnosis at index or within 12 months post index. Results were stratified by provider type (primary care physician (PCP)/specialist). A disjoint k-means cluster analysis identified treatment pathways using annual prescription numbers for CS, hydroxychloroquine (HCQ), mycophenolate mofetil, azathioprine, and methotrexate as input variables.

## Results

The study identified 2,086 newly diagnosed SLE patients (mean age: 47.2 years; female: 91%). In the 4 years post index, 1,031 (49.4%) patients were not actively treated (<0.05 prescriptions/year). Of the 219 (10.5%) patients who primarily received CS, 42 had persistently high numbers of prescriptions (~1/month), and 177 received 4.9 (mean) prescriptions in Year 1, decreasing in Years 2 to 4. Three subgroups emerged within the 606 (29.1%) patients who primarily received HCQ: persistent high number of prescriptions (~1/month), persistent moderate number of prescriptions (3.2 to 4.1/year), and poor adherence (Year 1, 8.7 prescriptions; Years 2 to 4, decreasing prescriptions). Both CS and HCQ were received by 138 (6.6%) patients; 56 had high numbers of prescriptions (Years 1 to 4); 82 showed progressively decreasing prescriptions. Fifty-four (2.6%) and 38 (1.8%) patients had moderate numbers of prescriptions for methotrexate (5.4 to 8.4/year) and azathioprine (5.7 to 7.5/year), respectively, with some CS and HCQ prescriptions. Treatment patterns differed in patients seen by specialists versus PCPs (*P *< 0.0001). Specialist-treated patients had a lower no-treatment rate than PCP-treated patients, and higher rates in active treatment clusters (Table 1).

**Table 1:** Longitudinal treatment patterns according to primary treatment by specialist or PCP

Cluster	Interpretation	Treated primarily by specialists^a^		Treated primarily by PCPs^a^	
		
		*n*	%	*n*	%
1	Not actively treated throughout (<0.05 (mean) annual prescriptions)	216	26.2	815	64.6
2	CS only: high number of prescriptions (chronic use)	29	3.5	13	1.0
3	CS only: moderate number of prescriptions with slow reduction	85	10.3	92	7.3
4	HCQ only: high number of prescriptions (chronic use)	102	12.4	56	4.4
5	HCQ only: moderate number of prescriptions (chronic use)	136	16.5	134	10.6
6	HCQ only: poor adherence	111	13.5	67	5.3
7	CS plus HCQ: high number of prescriptions (chronic use)	32	3.9	24	1.9
8	CS plus HCQ: poor adherence	51	6.2	31	2.5
9	Methotrexate: moderate number of prescriptions plus some prescriptions for CS and HCQ	34	4.1	20	1.6
10	Azathioprine: moderate number of prescriptions plus some prescriptions for CS and HCQ	28	3.4	10	0.8
Total		824	100.0	1262	100.0

^a^Patients who visited a specialist (including a rheumatologist, dermatologist, nephrologist, ophthalmologist, or oncologist) in >50% of SLE-related office visits were defined as primarily seen by specialists. Patients who visited PCPs in >50% of SLE-related office visits were defined as primarily seen by PCPs. The difference in treatment patterns between the two groups was statistically significant (*P *< 0.0001).

## Conclusions

Treatment patterns were observed among SLE patients using medical resources. In the 4 years post diagnosis: ~50% of patients were not actively treated; 50% received CS, HCQ, and immunosuppressants with differing combinations, intensities, and adherence levels. Specialists provided more intensive treatment than PCPs.

## Competing interests

CM, HK, JP, and SN are employees of and hold stock in GlaxoSmithKline. DJW is a consultant for GlaxoSmithKline.

